# On Skin Grafting

**Published:** 1873-11

**Authors:** J. T. Whitaker


### Our Exchanges.

*THE CLINIC:*

NOTE ON A CASE OF SKIN GRAFTING UPON AN ULCER OF THE LEG OF TWENTY YEARS STANDING.—COMPLETE CURE.—*J. T. Whitaker, M.D.* Skin Grafting upon the surface of ulcers, cicatrices, etc., has now become so common an operation that a case must present some extrinsic feature of interest to justify its publication. The duration of the disease and its extreme obstinacy to all forms of treatment are the features in the present instance.

Mrs. A. L. G., a lady of prominent social position in Covington, Ky., well advanced in life, had been afflicted with chronic ulcer of the leg for twenty years. Every possible variety of treatment had been adopted, with but temporary relief at best, during these two decades of years, until she finally lost all hope of permanent cure and was fain content to secure such alleviation as the domestic "salves" would afford. When pain became excessive, she had resort to anodynes, and upon one or two occasions, at least, had administered to her, by a physician, subcutaneous injections of morphia.

The ulcer, when I first saw it, was at its worst phase. It was about the size of the out-stretched hand, dipped so deeply into the tissues as to be but a line or two from the tibia and was surrounded, beyond its dull livid edge, with a broad zone of discolored integument of almost board-like hardness. The discharge of pus was excessive.

I snipped off a piece of skin from the integument of my own fore-arm about the size of the surface of a split pea; two similar pieces I took from her son-in-law, a healthy, vigorous adult, and one from the fore-arm of each of her two grand-children, all volunteers.

Having rolled out these pieces upon my thumb nail, I set them upon clean surfaces at diffierent parts of the ulcer, fastened them each with thin strips of adhesive plaster notched in over the grafts, for observation, and strapped the whole leg with crossed bands of plaster, from the ankle to the knee. By the third day all the grafts had adhered and lines of transculent tissue radiated from the collodion-like films, the appearance the grafts had now assumed, and the healing process was under full headway.

This process continued without hindrance up to the end of the third week, when the ulcer was entirely coated over. In another week the filmy surface had increased in depth to such degree as to resemble healthy skin. Simultaneously, the indurated tissue about the ulcer became more soft and pliable, and the patient could now use the limb in locomotion. As the skin over the whole leg continued tense and shiny, I directed her to wear, during the day, an elastic stocking of proper size, which, besides preventing recurrence of the ulcer, gives her great ease and comfort.

The exceptionally good result which I have had in this case, I attribute, in the main, to the good sense of my patient in religiously observing directions as to rest and ablutions after the adhesion of the grafts.

I used in all but four grafts, but it is generally advisable to employ a greater number. In this connection I extract from the *New York Record*, November 1, 1873, the method of Dr. Van Wagner, of Bellevue Hospital, who has set successively over two thousand grafts:

"The plan which he now follows, after trying all the plans of which he could find any account, in this: Take up a good thick fold of skin, and, from a smooth portion of this fold, shave a piece of skin, embracing in its thickness a few cells of the true skin, and somewhat variable in length and breadth. A good size is three-fourths of an inch in length and three-eighths of an inch in breadth.

"The ulcerated surface to which they are to be applied must have the shining glue-like appearance removed from the granulations, by careful wiping before the grafts are set. The grafts are then set about one inch from the peripheral edge of the ulcer, at intervals of one-half or three-quarters of an inch, forming a complete circle. As soon as set they are dressed with lint smeared with simple cerate, and over this a roller is applied. This dressing is to be removed the *next day* after the grafts are set, and be renewed every day. After the second or third day the ordinary strapping can be resorted to, and where the space between the circle of grafts and the peripheral edge of the ulcer becomes closed, another circle of grafts may be set, and so on until complete cicatrisation has been secured. If these rules are strictly adhered to, scarcely a graft is lost, the firmness and rapidity with which they adhere to the granulations sometimes being extremely marked."

I thought seriously at first of adopting Nussbaum's method of circumsision, and held it, indeed, in reserve as a dernier resort to which, however, I was fortunately not obliged to resort. Fortunately, in the sense that the more recent reports as to the efficacy of this method are not satisfactory, Dr. Bruen, Resident Physician at the Philadelphia Hospital reports (Phil. *Times*, Nov. 15, '73), two cases of abscess and ulcer formation in the line of incision. He says:

"It does not appear to me that cell-change is sufficiently active, nor is the vitality of chronic leg-ulcers of such a character as to justify the danger of enlarging the pathological surface when the morbid conditions can be treated by numerous other measures not open to such objections. It is proper to add that both these two cases seemed favorable for a fair trial of this new mode of treatment, as both men enjoyed excellent health.

